# Endothelial Expression of Endothelin Receptor A in the Systemic Capillary Leak Syndrome

**DOI:** 10.1371/journal.pone.0133266

**Published:** 2015-07-15

**Authors:** Albert C. Sek, Zhihui Xie, Kaoru Terai, Lauren M. Long, Celeste Nelson, Arkadiusz Z. Dudek, Kirk M. Druey

**Affiliations:** 1 National Institute of Allergy and Infectious Diseases, National Institutes of Health, Bethesda, MD, United States of America; 2 Division of Hematology/Oncology, Department of Medicine, University of Illinois at Chicago, Chicago, IL, United States of America; Hungarian Academy of Sciences, HUNGARY

## Abstract

Idiopathic systemic capillary leak syndrome (SCLS) is a rare and potentially fatal vascular disorder characterized by reversible bouts of hypotension and edema resulting from fluid and solute escape into soft tissues. Although spikes in permeability-inducing factors have been linked to acute SCLS flares, whether or not they act on an inherently dysfunctional endothelium is unknown. To assess the contribution of endothelial-intrinsic mechanisms in SCLS, we derived blood-outgrowth endothelial cells (BOEC) from patients and healthy controls and examined gene expression patterns. *Ednra*, encoding Endothelin receptor A (ETA)—the target of Endothelin 1 (ET-1)—was significantly increased in SCLS BOEC compared to healthy controls. Although vasoconstriction mediated by ET-1 through ETA activation on vascular smooth muscle cells has been well characterized, the expression and function of ETA receptors in endothelial cells (ECs) has not been described. To determine the role of ETA and its ligand ET-1 in SCLS, if any, we examined ET-1 levels in SCLS sera and functional effects of endothelial ETA expression. ETA overexpression in EAhy926 endothelioma cells led to ET-1-induced hyper-permeability through canonical mechanisms. Serum ET-1 levels were elevated in acute SCLS sera compared to remission and healthy control sera, suggesting a possible role for ET-1 and ETA in SCLS pathogenesis. However, although ET-1 alone did not induce hyper-permeability of patient-derived BOEC, an SCLS-related mediator (CXCL10) increased *Edrna* quantities in BOEC, suggesting a link between SCLS and endothelial ETA expression. These results demonstrate that ET-1 triggers classical mechanisms of vascular barrier dysfunction in ECs through ETA. Further studies of the ET-1-ETA axis in SCLS and in more common plasma leakage syndromes including sepsis and filovirus infection would advance our understanding of vascular integrity mechanisms and potentially uncover new treatment strategies.

## Introduction

The Systemic Capillary Leak Syndrome (SCLS) is a catastrophic orphan disease discovered by Clarkson in 1960 [1–3]. Patients experience transient—yet recurrent and highly unpredictable—episodes of hypotensive shock and peripheral edema resulting from plasma leakage into soft tissues. Episodes of hypotension, accompanied by hemoconcentration and hypoalbuminemia are required for the diagnosis. There are no specific biomarkers for SCLS, but 85–95% of patients have a monoclonal gammopathy of unknown significance (MGUS); its relationship to disease pathogenesis is unknown. Complications including rhabdomyolysis, renal failure, arterial and venous thrombosis, stroke, and compartment syndromes of the extremities may ensue—frequently requiring fasciotomies and occasionally leading to lasting neuromotor defects and/or loss of limbs. Treatment of acute flares of SCLS, including corticosteroids, antihistamines, or sympathomimetics, is primarily supportive. Prophylactic treatments have been developed by trial-and-error. Most recently, the use of intravenous immunoglobulins (IVIG) has emerged as a promising strategy to curtail recurrence of attacks [4–6]. 5-year mortality from SCLS in the absence of prophylactic treatment may be as high as 80% [5].

The pathogenesis of SCLS is unknown, and extremely low disease incidence (< 500 cases reported in the literature since its original description) has severely hampered research efforts [2,7]. We have accumulated one of the world’s largest registries of SCLS patients and launched systematic molecular biostudies. We reported that bloodborne factors known to promote vascular hyper-permeability—vascular endothelial growth factor (VEGF) and angiopoietin-2 (Angpt-2)—were increased in acute SCLS sera [8,9]. In addition, episodic—but not basal—SCLS sera induce a breakdown in vascular barrier function through actin cytoskeleton and adherens junctions remodeling in endothelial cells (ECs) [8,9].

Whether soluble blood mediators induce SCLS flares or act on an endothelium prone to exaggerated reactivity to permeability-inducing factors is unknown. In an effort to address the latter possibility, we generated ECs from peripheral blood of SCLS subjects and age-, sex-, and ethnicity-matched controls. These cells, termed blood-outgrowth, or late-outgrowth, endothelial cells (BOEC) are thought to originate from bone marrow-derived angioblasts of non-hematopoietic lineage. BOEC have the characteristic morphology and molecular signature of mature ECs and can promote blood vessel formation in vivo [10–12]. BOEC possess a stable phenotype that facilitates exploration of diseases associated with vascular abnormalities and their genetic underpinnings [13–15]. For example, BOEC have been used extensively to model endothelial function in vascular disorders such as sickle cell anemia [16] and Von Willebrand disease [17].

Here we studied BOEC from patients with SCLS and healthy controls. Gene expression analysis revealed upregulation of *Ednra*, the gene encoding Endothelin receptor A (ETA), in ECs from SCLS subjects compared to that in unaffected controls. Although ETA is well known to regulate vascular smooth muscle tone, its expression and potential functions in endothelial integrity have not been studied [18,19]. We show that ETA activation in ECs induces hyper-permeability, and we explore its potential role in vascular barrier defects that are the key feature of SCLS.

## Materials and Methods

### Patients

The diagnosis of acute systemic capillary leak syndrome was made in the context of one or more episodes of 1) hypotension, 2) elevated hematocrit, and 3) hypoalbuminemia, according to established criteria [5]. All patients were seen at the Clinical Center of the NIH. Written informed consent was obtained from each patient, and the study protocol (I-0184) conformed to ethical guidelines approved by the Institutional Review Board of NIAID, NIH. “Episodic” sera were collected during an SCLS flare requiring hospitalization or visit to a physician, generally within 24–48 hours of presentation. “Basal” sera were obtained during asymptomatic intervals of disease remission.

### Blood Outgrowth Endothelial Cells (BOEC)

Whole blood (100 ml) was collected from SCLS subjects during an asymptomatic interval or from age, sex, and ethnicity-matched healthy controls. We prepared buffy coat mononuclear cells from blood using Lympho separation medium (MP Biomedicals). Cells were re-suspended in Endothelial Growth Medium (EGM-2, Lonza), and plated into culture wells coated with collagen I. The culture medium was changed every other day for several weeks until the colonies formed, and BOEC were expanded according to published protocols [12,20].

### Gene expression analysis

Blood obtained from SCLS patients collected during an asymptomatic interval was used to derive BOEC. In tandem, blood samples from healthy donors were processed as outlined above for growth of control BOEC. We extracted RNA from low-passage cultured cells using the RNAeasy kit (Qiagen). Individual RNA samples were used to generate separate cDNA specimens using the SuperScript reverse transcriptase mix containing random hexamer oligonucleotides (Invitrogen). We generated pooled samples by mixing equal volumes of cDNA from each patient or healthy control. We analyzed gene expression in pooled disease or control samples containing equal amounts of cDNA from each subject by quantitative PCR array (Human Endothelial Cell Biology Profiler Array, Qiagen) (S1 Table). We validated expression of the most prominent differentially expressed transcripts in the array in individual cDNA samples by qPCR using gene-specific TaqMan probes (Applied BioSystems) according to the manufacturer’s guidelines. Catalog numbers for probes used are as follows: EDNRA: Hs03988672_m1; EDNRB: Hs00240747_m1; UBC: Hs00824723_m1; *IL1b*: Hs01555410_m1; *PF4*: Hs00427220_g1; *CX3CL1*: Hs00171086_m1.

### Cell culture, reagents, retroviral transduction

EAhy926 (EAhy) endothelioma cells (the gift of Dr. J. Silvio Gutkind, NIDCR) were cultured in DMEM supplemented with 10% FBS. Human primary dermal microvascular ECs (HMVEC) were obtained from Lonza; vascular (aortic) smooth muscle cells (VSMC) were purchased from the American Type Culture Collection (ATCC). Cells were grown in endothelial growth medium or smooth muscle specific medium, respectively (Lonza). We purchased endothelin-1 and human thrombin from Sigma. We achieved stable expression of ETA in endothelial cells by transducing EAhy926 cells with retrovirus encoding ETA-YFP (the kind gift of Dr. Takahiro Horinouchi) or GFP as a control, essentially as described previously [21]. EAhy926 cells expressing ETA/YFP were selected by puromycin resistance. GFP/YFP^+^ cells were sorted using a FACSAria II flow cytometer (BD Biosciences).

### Ca^2+^ mobilization

We measured intracellular Ca^2+^ using a Flexstation II automated fluorimeter (Molecular Devices) as described previously [22]. Cells were seeded in black, 96-well clear bottom plates (1.5 x 10^4^ cells/well), incubated at 37°C for 24 hr and starved overnight in serum-free EBM medium containing 0.2% BSA. Prior to addition of agonist, cells were incubated with a Ca^2+^ indicator dye (FLIPR Calcium 3 Assay, Molecular Devices) at 37°C for one hr. In some experiments, specific antagonists were added together with Ca^2+^ binding dye or pre-incubated with recombinant human CXCL10 (Biolegend, 40 ng/ml) for four hrs prior to measurement of Ca^2+^ concentrations. We prepared solutions containing agonists in a separate plate (5x concentration), which were added to cells robotically in the fluorimeter. Following agonist addition, Relative Fluorescence Units (RFU) were recorded every 1.5 seconds for 180 seconds. Each experiment represents the average of quadruplicate samples per condition. RFU values were normalized to the highest RFU value for each experiment and presented as the percent maximal value.

### Transwell Permeability Assay

We assessed permeability by the translocation of FITC-labeled dextran (40 kD, Invitrogen) across endothelial monolayers, as described [23]. We plated ECs (either EAhy or BOEC as indicated) in duplicate for each condition (200,000 cells/well) in collagen-coated 24-well PET Transwell inserts (3 μm, Corning Costar). Confluent monolayers were serum-starved in EBM containing 0.2% BSA for one hr prior to addition of ligand and fluorescent tracer. Serial aliquots of medium were removed from the bottom chamber and transferred to a separate 96 well plate for measurement of fluorescence (excitation 485, emission 525) using the Flex Station II apparatus.

### Immunocytochemistry and immunoblotting

We seeded cells (40,000 cells/well) onto collagen-coated chamber well glass slides (Lab-Tek) and serum-starved in EBM containing 0.2% BSA for 1 h prior to stimulation with agonist for the indicated time periods. We fixed cells in PBS containing 4% paraformaldehyde for 15 min followed by permeabilization in PBS containing 0.1% Triton X-100 for 15 min. We immunostained cells with anti-VE-Cadherin antibody (BD Biosciences, catalogue no. 555611, 1:100 dilution) overnight, followed by staining with secondary antibody (Goat anti-mouse IgG-Alexa 594 conjugate, Invitrogen) for one hr. We washed cells several times with PBS prior to mounting on glass cover slips using ProLong Gold anti-fade reagent containing DAPI (Invitrogen). In some experiments, cells were incubated with Texas Red-conjugated phalloidin (Invitrogen, 0.8 U/well) for 20 min. We visualized cells using a Leica SP5 X-WLL confocal microscope.

### ET-1 ELISA

ET-1 levels in sera from patients with SCLS or healthy donors were measured by ELISA (R & D Systems) according to manufacturer’s guidelines.

### Statistical analysis

GraphPad Prism software was used for analysis. Data are presented as mean ± S.E.M. unless otherwise noted. Differences between means were tested by *t* test or nonparametric Mann-Whitney test for two groups or one- or two-way ANOVA for multiple groups. *P* values < 0.05 were considered significant.

## Results

### Characterization of BOEC morphology and gene expression

**Fig 1:**
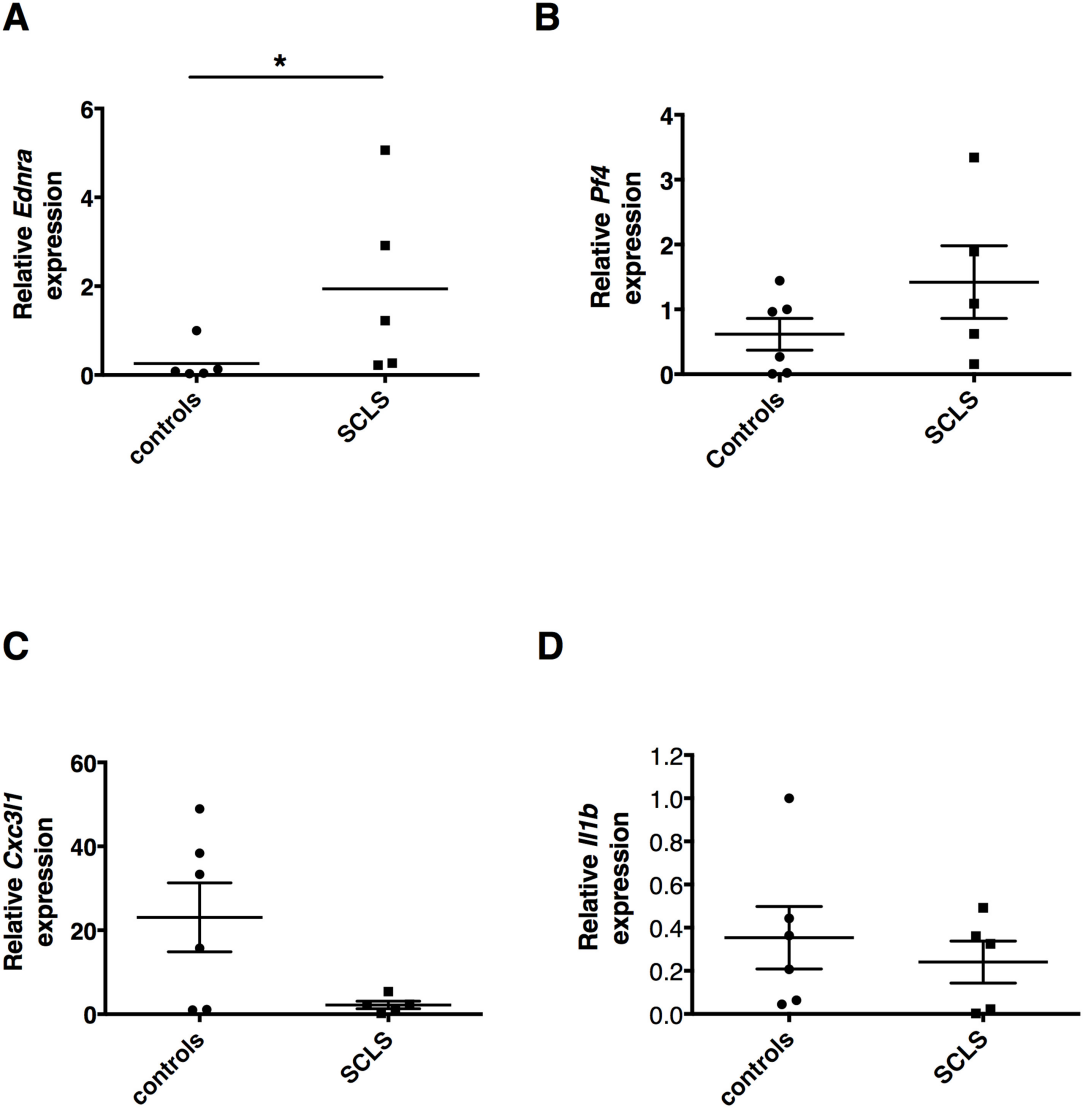
Morphology and gene expression in BOEC from SCLS patients and controls. (A) BOEC were expanded from peripheral blood. Image shows a representative EC monolayer immunostained with anti-VE-cadherin (green), F-actin (red), and DAPI (blue, nuclei). (B) Volcano plot of real-time qPCR array showing relative gene expression in SCLS BOEC relative to control on *x* axis and log *p* value on *y* axis.

To explore the role of endothelium in spells of profound vascular leakage that are the signal characteristic of SCLS, we generated BOEC from six subjects with classic acute SCLS and six unaffected healthy controls of similar age and ethnicity. Demographics and disease features of the patient cohort are shown in Table 1. Colonies of cells with characteristic cobblestone morphology and expression of the endothelial-specific surface marker VE-cadherin appeared in culture after several weeks (Fig 1A). We did not observe substantive differences in the number of colonies or in growth rates between cells from patients with SCLS versus those from healthy controls (data not shown) [12,24].

**Table 1:** Characteristics of study population. Demographics of SCLS patients and healthy controls from which BOEC were derived.

	Disease (n = 6)	Controls (n = 6)
**Males**	3	4
**Females**	3	2
**Mean age** (years ± S.D.)	46.6 ± 6	47 ± 15
**Caucasian**	5	4
**Classic acute SCLS**	6	NA
**MGUS**	6	0

We analyzed expression of 84 genes related to EC structure and function in cDNA samples pooled from BOEC of SCLS patients or healthy controls (n = 6/group) using a real-time qPCR array (Table 2 and S1 Table). Two genes (*Ednra*, *Pf4*) showed significantly increased expression (~13-fold and ~21-fold, respectively; *P* < 0.02) in SCLS BOEC compared to control whereas seven genes were downregulated more than two-fold and reached statistical significance (*Scl7a13*, *Bcl2*, *Mmp1*, *Tnf*, *Il1b*, *Cx3cl1*; *P* < 0.01) (Fig 1B). Among the most highly increased transcripts, *Edrna* encodes the endothelin receptor A (ETA)—a G-protein coupled receptor mediating Ca^2+^-dependent vascular smooth muscle contraction—whose expression has not been reported in endothelium [25–27]. *Pf4* encodes platelet factor 4 (PF4), a heparin-binding anti-angiogenic protein also secreted by activated platelets [28,29].

**Table 2:** Highly differentially expressed genes in SCLS BOEC v. controls. Gene expression in BOECs from SCLS patients and controls evaluated by qPCR array. Cycle threshold (Ct) values and fold-change for SCLS compared to controls for top differentially expressed genes.

Gene Symbol	C_t_ SCLS	Ct controls	Fold Control	*p* value*
*Ednra*	31.54	35.23	12.90	0.03
*Pf4*	30.26	34.71	21.81	0.00009
*Agt*	36.08	34.46	-3.06	0.001
*Bcl2*	32.80	31.03	-3.41	0.00003
*Mmp1*	21.47	19.22	-4.76	0.01
*Tnf*	31.69	29.37	-4.97	0.004
*Il1b*	32.30	29.92	-5.19	0.00001
*Cxc3l1*	29.75	27.13	-6.15	0.001
*Nppb*	39.85	36.17	-12.79	0.00007

*t test.

**Fig 2:**
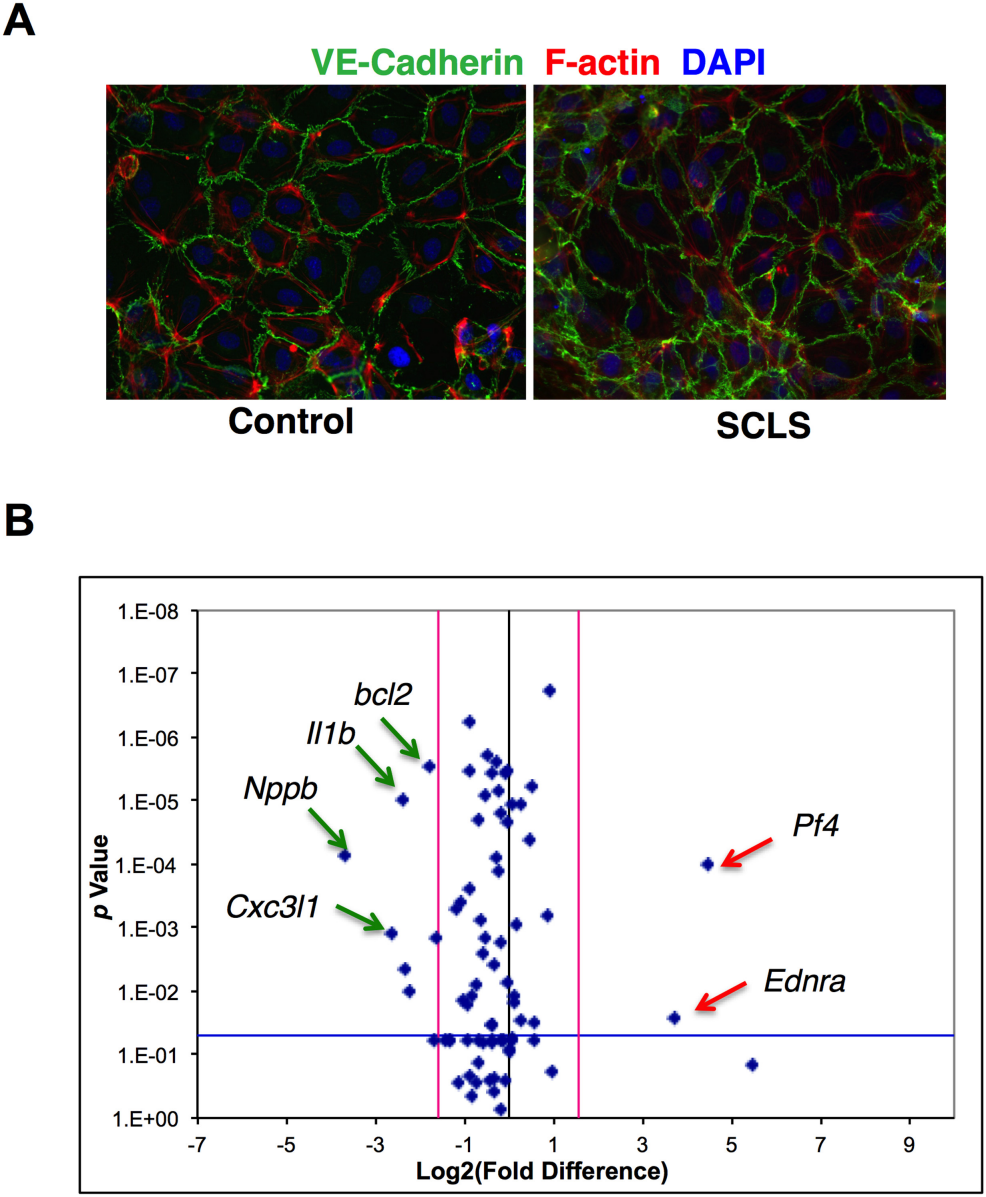
Gene expression in BOEC from individual subjects. (A-D) Relative expression of *Ednra*, *Cx3cl1*, *Pf4*, and *Il1b* in individual subjects as determined by real-time qPCR. *p = 0.03, Mann-Whitney test.

To determine the significance of these results in samples from individual subjects, we evaluated expression of the most highly differentially expressed genes from the array by real-time qPCR. Expression of *Ednra* was significantly increased in SCLS BOEC compared to control, whereas *Pf4*, *Cxc3l1*, and *Il1b* expression did not differ significantly between the two groups (Fig 2A–2D). We were unable to reliably evaluate ETA protein expression in these cells by either immunoblotting or immunofluorescence using five commercially available anti-ETA antibodies or an additional polyclonal antibody generated in-house. None of the antibodies detected endogenous ETA protein reproducibly in vascular smooth muscle cells (VSMC), which are known to express abundant ETA.

### ETA activation induces Ca^2+^ signaling in endothelial cells

**Fig 3:**
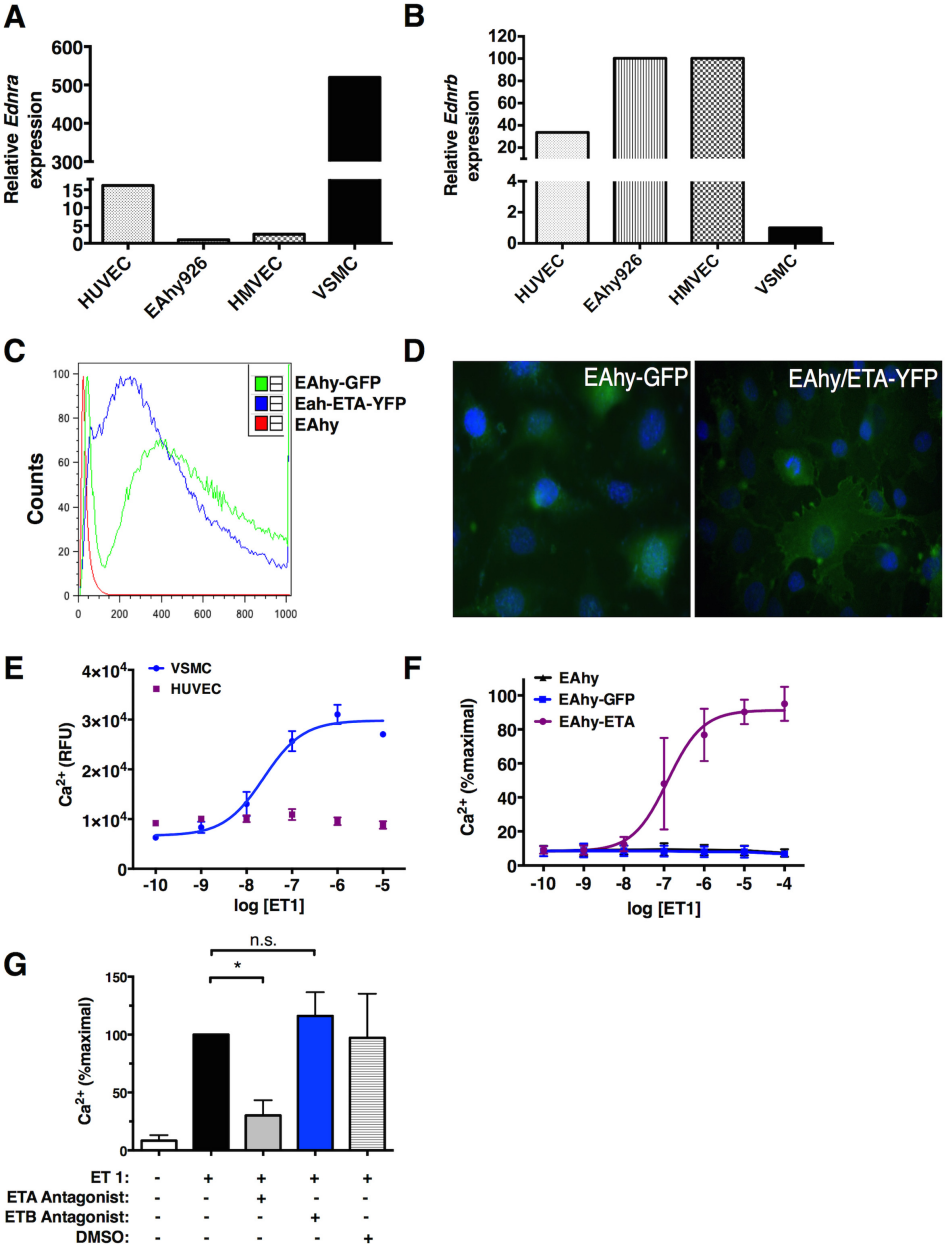
Expression and function of ETA receptors in endothelial cells. (A-B) Relative *Ednra* and *Ednrb* expression in indicated cell lines was determined by qPCR relative to expression in EAhy cells (A) or vascular smooth muscle cells (B) set as ‘1’. (C) EAhy cells were transduced with ETA-YFP or GFP retroviruses followed by selection of flow sorting; relative fluorescence post-sort was determined by flow cytometry. (D) Expression and localization of ETA-YFP or GFP was examined by fluorescence microscopy. (E-G) Ca^2+^ concentrations were measured in the indicated cell lines following treatment with various concentrations of ET-1 by fluorimetry. In (F) cells were pre-incubated with the indicated receptor antagonists or vehicle alone as a control for one hour prior to stimulation with ET-1 (200 nM). Data are mean ± S.E.M. of 3–4 individual experiments; *p = 0.04, one-way ANOVA.

Endothelium typically expresses ETB but not ETA receptors under homeostatic conditions [19]. *Edrna* expression was minimal in several EC lines commonly used to interrogate vascular functions including primary human dermal microvascular endothelial cells (HMVEC), human umbilical vein endothelial cells (HUVEC), or the HUVEC-derived immortalized endothelioma line EAhy926 (EAhy) whereas primary vascular smooth muscle cells expressed abundant *Ednra* (Fig 3A). In contrast, EC lines expressed readily detectable *Edrnb* (which encodes endothelin receptor B [ETB]) while VSMC did not (Fig 3B). To appraise the functional significance of endothelial *Ednra* expression for vascular integrity, we generated an EC line expressing ETA. We stably transfected EAhy cells with retrovirus encoding ETA-YFP or with GFP as a control; GFP/YFP^hi^ cells were sorted using flow cytometry and expanded for further analysis (Fig 3C). ETA-YFP localized both at the plasma membrane and cytosol of the cells by fluorescence microscopy as previously reported [21] (Fig 3D).

As expected, stimulation of VSMC with ET-1 induced prominent spikes in intracellular Ca^2+^ (Fig 3E) [25–27]. Treatment of ECs expressing ETA (EAhy-ETA) with ET-1 also evoked strong Ca^2+^ mobilization that was not seen in parental (EAhy926) or control (EAhy-GFP) cell lines (Fig 3F). Pretreatment of cells with ETA-specific antagonists prior to stimulation nearly abolished the Ca^2+^ response to ET-1 whereas an ETB-targeted antagonist had no effect (Fig 3G). These results demonstrate that ETA, but not ETB, mediates ET-1-induced Ca^2+^ signaling in ECs, suggesting that ETA expression in ECs confers a VSMC-like contractile phenotype in response to ET-1.

### ETA overexpression elicits endothelial hyper-permeability

**Fig 4:**
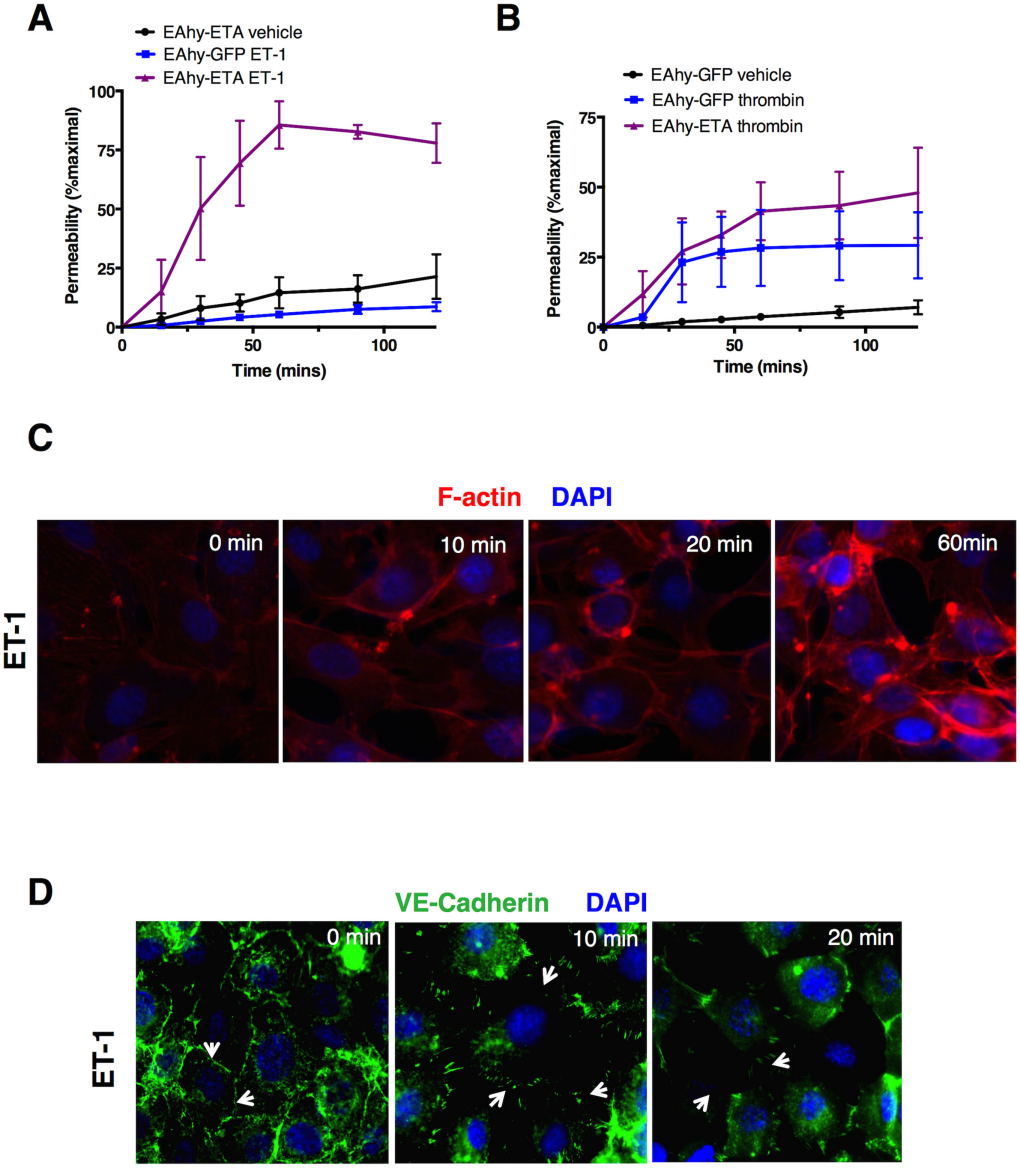
ETA mediates endothelial hyper-permeability. (A-B) Translocation of FITC-albumin across EAhy monolayers plated in duplicate in Transwell inserts was monitored over time following treatment with vehicle alone, ET-1 (1 μM, A) thrombin (2 U/ml, B). Data are mean ± S.E.M. of 2–3 individual experiments. (C-D) Cells were stimulated with thrombin or ET-1 for the indicated times followed by fixation and immunostaining with phalloidin (F-actin, red, C) or VE-cadherin antibody (red, D). Images are from a single experiment representative of 3 similar experiments.

To determine how ET-1 affects endothelial barrier defense mechanisms, we tracked the movement of FITC-albumin across endothelial monolayers plated in Transwell inserts over time. Compared to untreated cells, we observed significantly increased translocation of fluorescent tracer in EAhy-ETA cells treated with ET-1 compared to cells treated with vehicle alone or control EAhy-GFP cells treated with ET-1, with peak translocation at ~60 minutes (Fig 4A). Thrombin elicited comparable permeability responses in both cell lines (Fig 4B).

ET-1 evokes VSMC contraction by stimulating actin polymerization and myosin light chain kinase (MLCK) phosphorylation, which facilitates actin-myosin crossbridging [19]. GPCR ligands mediating vascular barrier disruption (e.g histamine, leukotrienes) evoke paracellular permeability by EC retraction through this pathway [30]. Exposure of EAhy-ETA cells to ET-1 similarly increased F-actin content, stress fibers, and induced physical disruption of the endothelial monolayer (Fig 4C). Virtually all known mediators of vascular hyperpermeability reduce surface expression of VE-cadherin [30], a key component of intercellular adherens junctions. ET-1 elicited starkly reduced membrane expression and junctional localization of VE-cadherin in EAhy-ETA but not control cells, with nearly absent surface expression 20 minutes after addition of the ligand (Fig 4D). These results indicate that ET-1 activation of ETA receptors de-stabilizes vascular integrity through canonical mechanisms including cell contraction and disruption of adherens junctions between adjacent ECs.

### Functional responses of BOEC

**Fig 5:**
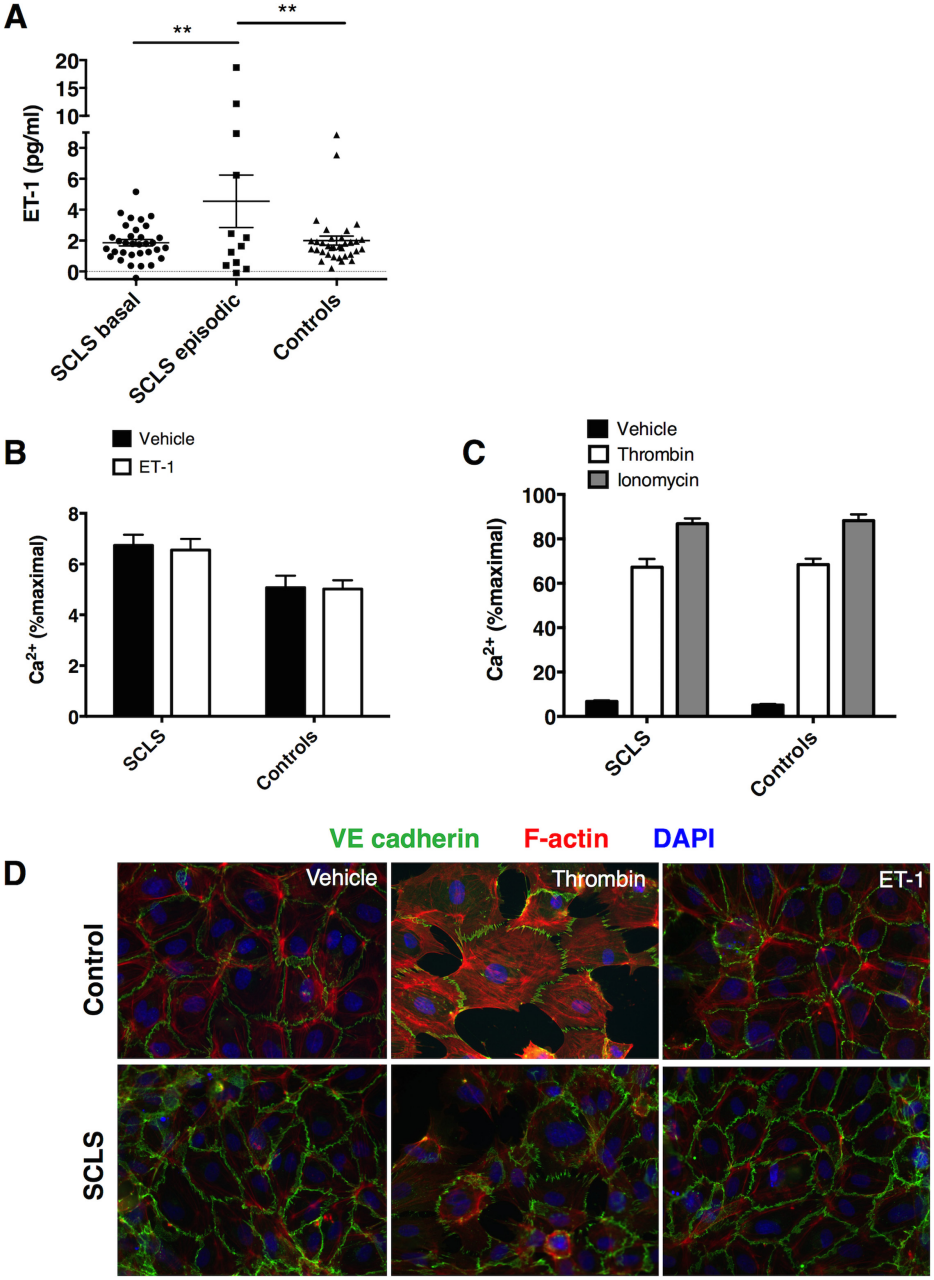
ETA-endothelin-1 axis in SCLS. (A) Serum ET-1 levels in SCLS sera (basal or acute) or healthy control sera were determined by ELISA. **p = 0.01, one-way ANOVA. (B-C) Intracellular Ca^2+^ concentrations were determined by fluorimetry as in Fig 3 in BOEC left untreated or stimulated with thrombin (2 U/ml) or ionomycin (1 μM) (B) or), ET-1 (2 μM). Data are mean ± S.E.M. of quadruplicate measurements from a single experiment representative of 3 similar experiments. (D) BOEC were stimulated with thrombin (2 U/ml) or ET-1 (2 **μ**M) for 20 min followed by fixation and staining with phalloidin (F-actin, red), anti-VE-cadherin (green), and DAPI (blue). Images are from a single experiment representative of 3 similar experiments using cells from different donors.

We next analyzed endothelin circuitry in SCLS. Transient surges in angiogenic and proinflammatory factors including VEGF, Angpt-2, TNFα CCL2, IL-1β and CXCL10 accompany flares of the disease [8,9]. Serum ET-1 levels in episodic SCLS sera were also significantly higher than those in SCLS pre-disease sera or healthy controls (Fig 5A). SCLS and control BOEC exhibited comparable intracellular Ca^2+^ flux in response to thrombin or Ca^2+^ ionophore stimulation (Fig 5B). Surprisingly, however, neither BOEC from SCLS subjects nor controls mobilized Ca^2+^ following treatment with ET-1 (at concentrations up to 20 μM—which induced maximal responses in EAhy cells) (Fig 5C). Thrombin strongly reduced surface VE-cadherin expression and elicited actin stress fiber formation and prominent intercellular gaps in confluent monolayers of either control or SCLS BOEC (Fig 5D). However, neither of these cells underwent morphologic changes or exhibited evidence of adherens junction remodeling in response to ET-1 stimulation.

### Proinflammatory SCLS mediators induce ETA expression

**Fig 6:**
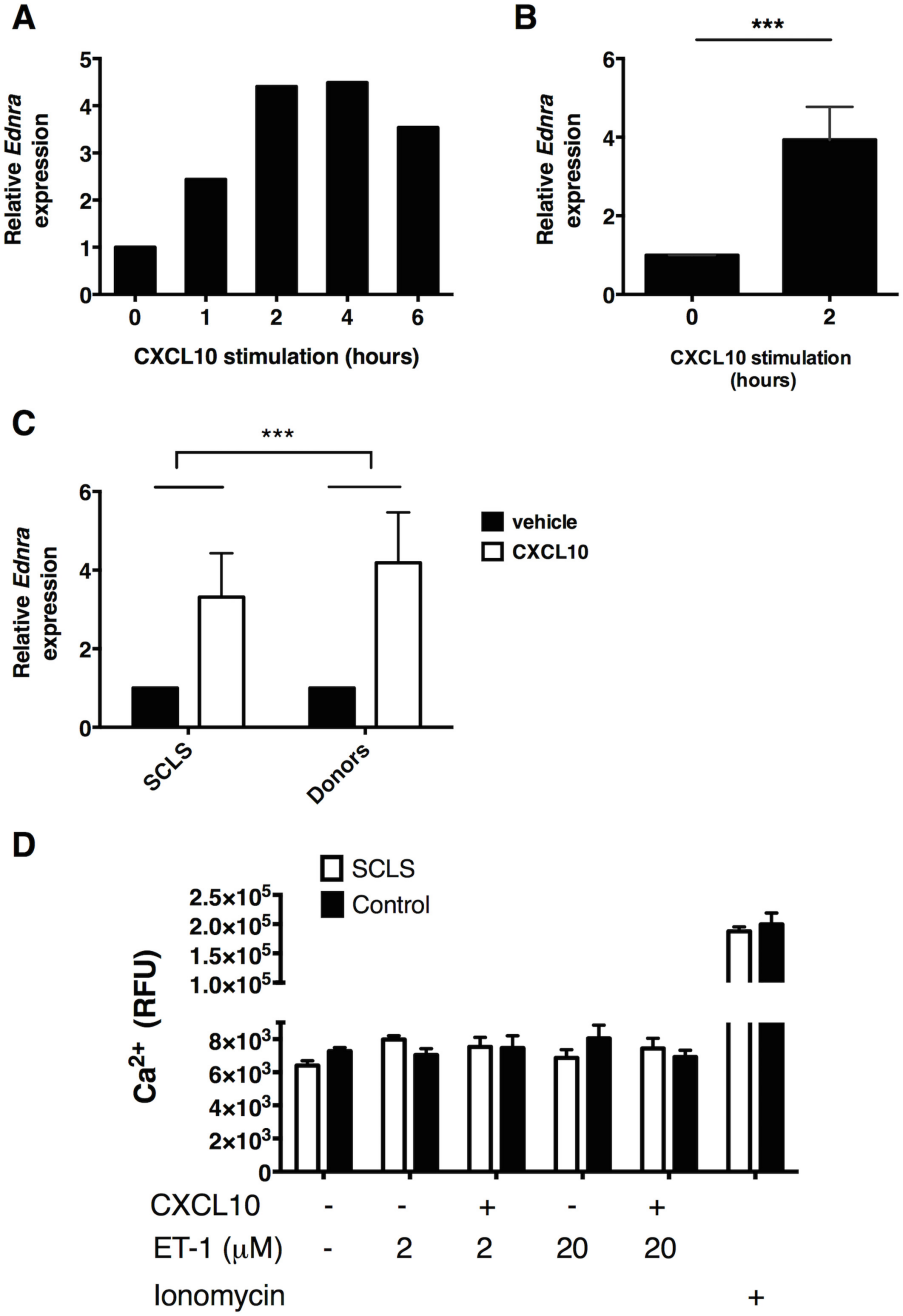
SCLS-associated mediator CXCL10 induce *Ednra* expression in endothelial cells. (A-B) EAhy cells were left untreated (0 h) or stimulated with CXCL10 (40 ng/ml) for the indicated times followed analysis of *Ednra* expression by qPCR. Data are average of duplicate measurements from a single experiment (A) or mean ± S.E.M of four individual experiments (B); ***p = 0.0004; *t* test. (C) *Edrna* expression in BOEC from SCLS patients or controls (n = 4/group) left untreated or stimulated with CXCL10 (40 ng/ml) for 4 hr was determined by qPCR. Data are mean ± S.E.M.; ***p = 0.005, two-way-ANOVA. (D) BOEC from a subject with SCLS or healthy donor were pretreated with CXCL10 (40 ng/ml) for four hours prior to stimulation with the indicated concentrations of ET-1 or ionomycin and measurement of intracellular Ca^2+^ concentrations by fluorimetry. Bar graph is mean ± S.E.M. of quadruplicate values from a single experiment representative of 3 similar experiments.

The unexpected unresponsiveness of SCLS BOEC to ET-1 led us to hypothesize that ETA expression in BOEC in vitro may be inadequate to induce easily measurable responses in our assays. We hypothesized that ETA expression in SCLS ECs exposed to disease-related bloodborne factors in vivo could be higher than what we detect in standard culture conditions. To test this hypothesis, we first stimulated EAhy cells with putative SCLS mediators VEGF, Angpt-2, or CXCL10 and measured relative *Ednra* expression by real-time qPCR. Neither VEGF nor Angpt2 alone significantly affected expression of *Ednra* (data not shown); in contrast, CXCL10 induced robust increases in *Edrna* expression beginning an hour after stimulation and persisting for up to six hours (Fig 6A). *Edrna* expression increased nearly four-fold after four hours of CXCL10 treatment compared to untreated cells (Fig 6B). In both SCLS and control BOEC, CXCL10 similarly augmented *Edrna* expression after two hours (Fig 6C). Pretreatment of BOEC with CXCL10 did not elicit Ca^2+^ responses to ET-1 (Fig 6D). Nonetheless, the marked upregulation of *Ednra* expression induced by an SCLS-associated proinflammatory mediator suggest that further studies of ETA in the pathogenesis of acute SCLS flares are warranted.

## Discussion

ET-1’s control of blood vessel functions reflects a balance between its opposing actions on vascular smooth muscle and endothelium—potent constriction of the former through ETA-mediated Ca^2+^ signaling and relaxation-promoting activities of the latter through ETB-elicited nitric oxide (NO) production [19]. Perturbations of the ETA-ETB axis have been implicated in atherosclerosis and pulmonary artery hypertension, and ET receptor antagonists have been used successfully to treat both essential and pulmonary hypertension [18]. In animal models of acute lung injury (ALI), tenzosentan—a nonselective antagonist of both ETA and ETB—attenuated sepsis-associated microvascular permeability in the lung. However, this appeared to be due to reduced pulmonary arterial pressures, which is most likely mediated by ETA expressed on smooth muscle cells, and no data distinguished which ET-1 receptor on endothelium was responsible for this effect [31,32].

Here we detected increased levels of ET-1 in acute SCLS sera and enhanced expression of ETA in ECs from patients with SCLS, suggesting a potential role for ET-1 and ETA in the recurrent episodes of overwhelming breakdowns in endothelial barrier defense that characterize this rare vascular disorder. A limitation of our study is that acute serum samples were acquired off-site because the catastrophic nature of acute bouts of SCLS precludes patient transport. We know that cytokine levels (e.g. VEGF and Angpt-2) vary considerably throughout the course of acute SCLS flares, suggesting that we may have been unable to capture peak ET-1 levels in some patients (6). To our knowledge, ETA expression on microvascular ECs has not been described, nor has the role of ETA in vascular integrity been explored previously. We demonstrated that activation of overexpressed ETA in the EC line EAhy by ET-1 elicits Ca^2+^ mobilization, actin cytoskeletal rearrangements, and adherens junction remodeling (Fig 5), suggesting that ETA expression in ECs may generate endothelial hyper-permeability under certain circumstances.

Few studies have detected ETA expression in ECs in vivo. Analysis of kidney cells from *Edrna* ^*EGFP/+*^ knockin mice showed expression of EGFP limited to CD31 (platelet-EC adhesion molecule 1 [PECAM-1])-α smooth muscle actin^+^ cell populations—markers of ECs and VSMC, respectively. These findings correlated with *Ednra* mRNA expression patterns, indicating absent ETA expression in renal ECs under homeostatic conditions [33]. In a separate study, ETA expression was found to be upregulated in mouse renal glomerular endothelial cells (RGEC) following ischemia/reperfusion injury and in human RGEC subsequent to hypoxic insult or exposure to TNFα or IL-6 [34]. Aveanian et al. observed ETA expression in the nucleus of human aortic vascular endothelial cells (hVECs), and stimulation of hVECs in vitro elicited Ca^2+^ mobilization at concentrations similar to those used in our study (1 μM) [35]. However, although ET-1 stimulation of hVEC monolayers induced hyper-permeability as evidenced by reduced transendothelial electrical resistance (TEER) and internalization of gap junction proteins between adjacent cells, the authors did not establish which receptor (ETA or ETB) mediated these events.

Because SCLS BOEC did not respond to ET-1 in our functional assays (Ca^2+^ mobilization, cell morphology) but exhibited reactivity to thrombin comparable to controls, SCLS EC reactivity might be limited to specific permeability triggers, rather than indicative of a global defect. Second, ETA quantities in BOEC may inadequately reflect its expression in microvasculature in vivo. We have already demonstrated that factors present in acute SCLS sera (e.g. CXCL10) further augment ETA expression in EC lines and BOEC (Fig 6). Profound hypotension, organ hypoperfusion, and a cascade of proinflammatory mediators (including IL-6 and TNFα) are also characteristic of acute SCLS flares [9], suggesting additional routes of ETA upregulation (as discussed above). Therefore, BOEC studied here under basal conditions may not fully recapitulate the phenotype of ECs during full-blown episodes of SCLS. Third, it is possible that increased ETA expression could be selectively upregulated in vivo in specific vascular beds. BOEC are derived without selection for markers of specific blood vessels—Duffy antigen for chemokines (DARC), a marker for post-capillary venules, is an example [36]—which could result in a mixed EC population of diverse origins following expansion in culture.

Nonetheless, the disparate gene expression patterns of SCLS and control BOEC cultured outside the context of intact blood vessels support the possibility of intrinsic, possibly heritable defects in endothelial barrier defense mechanisms. Although no consistent familial aggregations are apparent in SCLS [3,37], our GWAS results of twelve patients strongly suggest a genetic component. Odds ratios (7–41) and *p* values for the top SCLS-associated variants (8×10^−4^ and 4x10^-6^) were outsized for this small cohort, implying high disease allele penetrance [37]. Because SCLS occurs rarely and sporadically, de novo mutations could also account for the phenotype. Genetic analysis of the putative target tissue (i.e. ECs) may be required to discover spontaneous and/or low frequency somatic mutations. Future studies of SCLS cells and tissues—ECs derived from pluripotent stem cells (iPS-ECs), for example—should allow us to differentiate among these possibilities. More broadly, our results provide a rationale for the study of endothelial ETA expression and function in more common disorders associated with plasma leakage including sepsis or infections with Dengue or Ebola viruses.

## Supporting Information

S1 Table Gene expression analysis by qPCR array. Full list of genes whose expression was analyzed in BOEC RNA. Results categorized by the following: **Fold-Change** (2^(- Delta Delta Ct)) is the normalized gene expression (2^(- Delta Ct)) in the Test Sample divided the normalized gene expression (2^(- Delta Ct)) in the Control Sample. **Fold-Regulation** represents fold-change results in SCLS samples versus controls. Fold-change and fold-regulation values greater than 2 are indicated in red; fold-change values less than 0.5 and fold-regulation values less than -2 are indicated in blue. **p-values:** The p values are calculated based on a Student’s t-test of the replicate 2^(- Delta Ct) values for each gene in the control group and treatment groups, and p values less than 0.05 are indicated in red. **Comments: A**: This gene’s average threshold cycle is relatively high (> 30) in either the control or the test sample, and is reasonably low in the other sample (< 30). These data mean that the gene’s expression is relatively low in one sample and reasonably detected in the other sample suggesting that the actual fold-change value is at least as large as the calculated and reported fold-change result. This fold-change result may also have greater variations if p value > 0.05; therefore, it is important to have a sufficient number of biological replicates to validate the result for this gene. **B**: This gene’s average threshold cycle is relatively high (> 30), meaning that its relative expression level is low, in both control and test samples, and the p-value for the fold-change is either unavailable or relatively high (p > 0.05). **C**: This gene’s average threshold cycle is either not determined or greater than the defined cut-off (default 35), in both samples meaning that its expression was undetected, making this fold-change result erroneous and un-interpretable.
(XLSX)
