# Correction: Endothelial Expression of Endothelin Receptor A in the Systemic Capillary Leak Syndrome

**DOI:** 10.1371/journal.pone.0137373

**Published:** 2015-09-01

**Authors:** Albert C. Sek, Zhihui Xie, Kaoru Terai, Lauren M. Long, Celeste Nelson, Arkadiusz Z. Dudek, Kirk M. Druey

The captions for Figs 1 and 2 are switched. The caption that appears with Fig 1 corresponds to Fig 2, and the caption that appears with Fig 2 corresponds to Fig 1. Please see the figures with the appropriate captions below.

**Fig 1:**
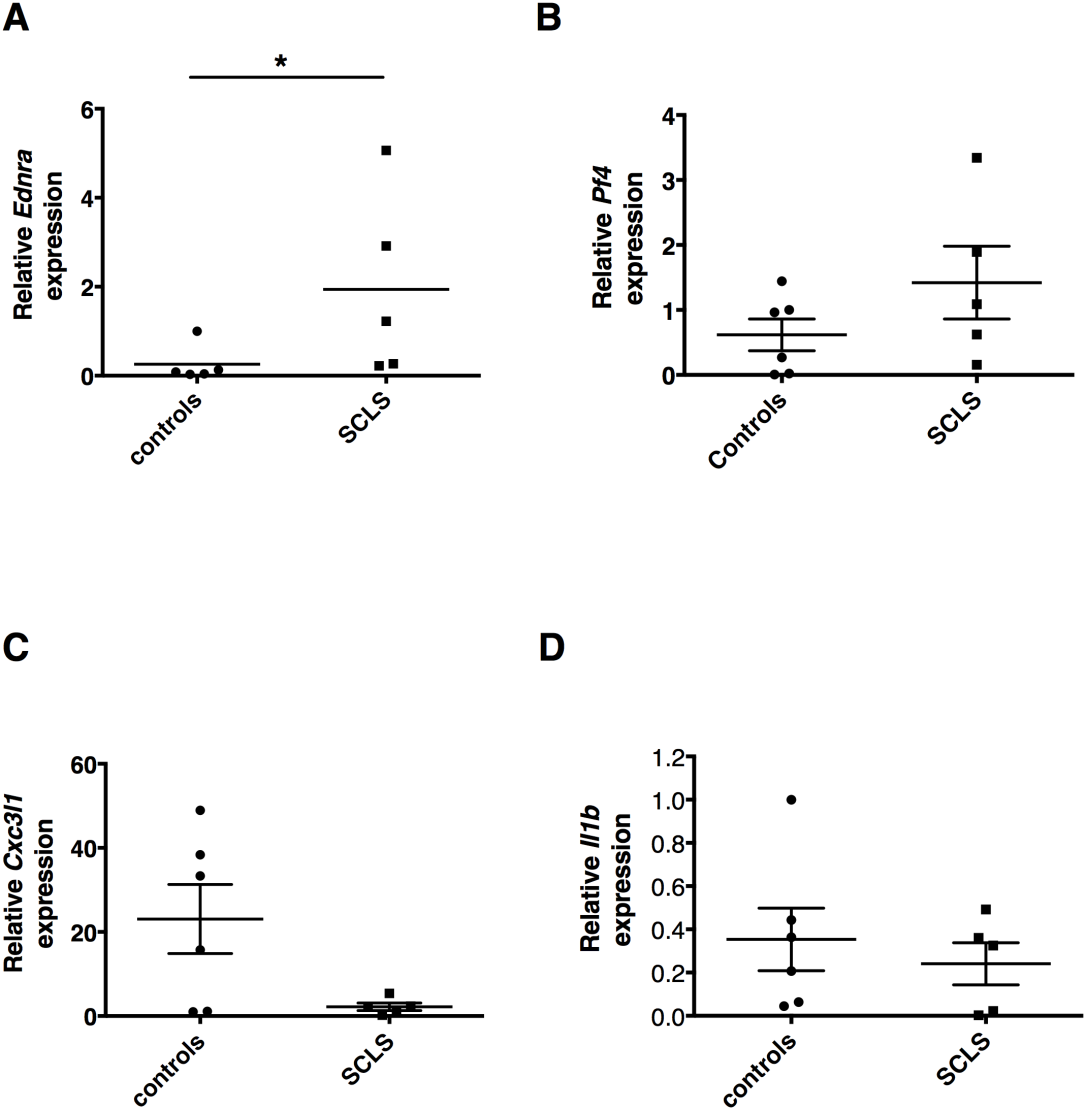
Gene expression in BOEC from individual subjects. (A-D) Relative expression of *Ednra*, *Cx3cl1*, *Pf4*, and *Il1b*in individual subjects as determined by real-time qPCR. *p = 0.03, Mann-Whitney test.

**Fig 2:**
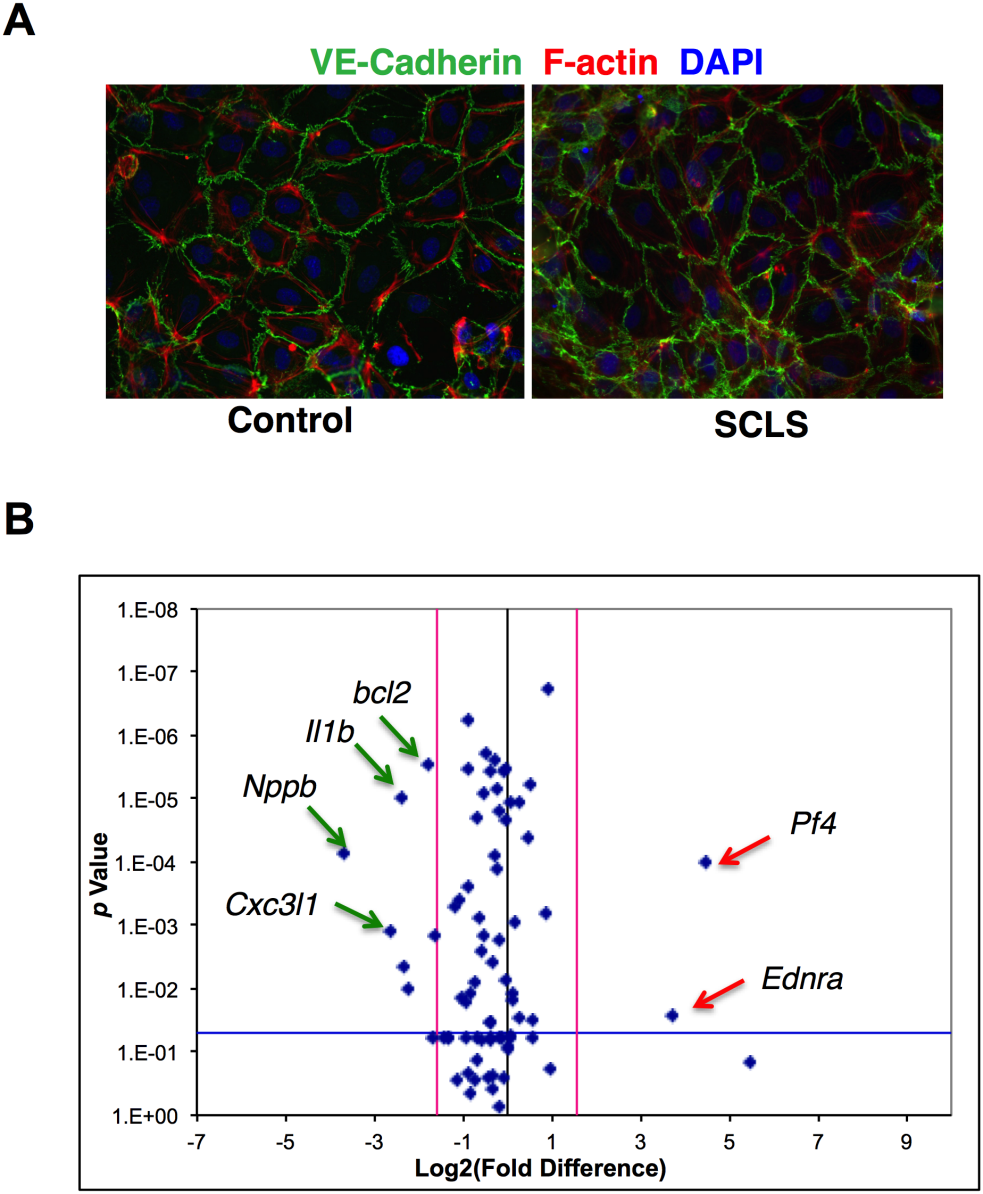
Morphology and gene expression in BOEC from SCLS patients and controls. (A) BOEC were expanded from peripheral blood. Image shows a representative EC monolayer immunostained with anti-VE-cadherin (green), F-actin (red), and DAPI (blue, nuclei). (B) Volcano plot of real-time qPCR array showing relative gene expression in SCLS BOEC relative to control on *x* axis and log *p*value on *y* axis.
